# Use of the kidney injury molecule-1 as a biomarker for early detection of renal tubular dysfunction in a population chronically exposed to cadmium in the environment

**DOI:** 10.1186/2193-1801-2-533

**Published:** 2013-10-17

**Authors:** Werawan Ruangyuttikarn, Amnart Panyamoon, Kowit Nambunmee, Ryumon Honda, Witaya Swaddiwudhipong, Muneko Nishijo

**Affiliations:** Division of Toxicology, Faculty of Medicine, Chiang Mai University, Chiang Mai, Thailand; School of Health Science, Mae Fah Luang University, Chiang Rai, Thailand; Department of Social and Environmental Health, Kanazawa Medical University, Ishikawa, Japan; Department of Social Medicine, Mae Sot General Hospital, Tak Province, Thailand; Department of Public Health, Kanazawa Medical University, Uchinada, Ishikawa, Japan

**Keywords:** KIM-1, Biomarker, Cadmium, Chronic exposure, Renal tubular dysfunction

## Abstract

Cadmium (Cd) has been found as an environmental pollutant in Mae Sot district, Tak province, Thailand. Prolong exposure to high levels of Cd of the resident increases high risk of Cd toxicity especially to kidney which is the primary target of Cd. In order to investigate the early effect of Cd induced renal dysfunction, a kidney injury molecule-1 (KIM-1), a novel biomarker of renal tubular dysfunction, was measured using an enzyme linked immunosorbent assay (ELISA). The method was validated and used to quantify the KIM-1 concentrations in the urine of 700 subjects (260 men, 440 women) who lived in the Cd contaminated area. The KIM-1 concentrations were compared to the concentrations of two conventional renal tubular dysfunction biomarkers, N-acetyl-β-D-glucosaminidase (NAG) and β_2_-microglobulin (β_2_-MG). Urinary KIM-1 was correlated with urinary and blood Cd as well as NAG. After adjustment of age and smoking, urinary KIM-1 was correlated with blood Cd more than urinary NAG did. Clear dose response relationships of urinary KIM-1 with urinary Cd were shown in both men and women. These results indicate that the urinary KIM-1 might be more sensitive biomarker than urinary NAG and β_2_-MG for an early detection of renal tubular dysfunction. It is useful as a tool to detect renal effect of toxicity due to chronic Cd exposure at high level.

## Introduction

KIM-1 is a type 1 cell membrane glycoprotein containing six-cysteine immunoglobulin-like and mucin domains (Ichimura et al. 1998). It has been reported as an undetectable biomarker in normal urine but after kidney injury is expressed at high levels (Ichimura et al. 2004; Vaidya et al. 2008; Ferguson et al. 2008). Elevated KIM-1 level was also found in human renal diseases associated with renal fibrosis, inflammation and dysfunction (van Timmeren et al. 2007). It was proposed as an early indicator of acute kidney injury over the conventional biomarker such as blood urea nitrogen, serum creatinine (Cr), urinary albumin, low molecular weight excreted proteins (α_1_- and β_2_-microglobulins (MG)) and tubular enzymes N-acetyl-β-D-glucosaminidase (NAG) (Han et al. 2002; Vaidya et al. 2006 Bonventre 2009,2010). It was also proposed as a novel urinary biomarker of cadmium induced nephrotoxicity via experimented rats and suggested that it could be indicated as a renal biomarker that is more sensitive and accurate than the other biomarkers (Vaidya et al. 2006; Prozialect et al. Prozialect et al. 2009). However, there is very limited report of KIM-1 study in human population exposed to cadmium (Cd) except the study of association between KIM-1 and urinary Cd in elderly with low-dose Cd exposure (Pennemans et al. 2011).

Cd is an important industrial and environmental pollutant that adversely affects multiple organ systems (WHO 1992; Jarup et al. 1998), of which the kidney is the primary target in humans with chronic Cd exposure (Friberg 1984; Lauwerys et al. 1984; Jarup et al. 1998). Impairment in renal tubular function, generalized osteomalacia and osteoporosis with the resultant multiple bone fractures are predominant clinical signs of the Itai-itai disease. Itai-itai disease was the most severe manifestation of cadmium toxicity in patients documented in Japan who suffered chronic exposure to high cadmium concentrations in contaminated water and rice (Nogawa et al. 1987; Aoshima et al. 1988).

In Thailand, elevated levels of Cd in paddy soils and rice grain downstream of a zinc mineralized area in Mae Sot district, Tak province was reported in 2005 (Simmons et al. 2005). The Mae Sot district is located in a mountainous area on the Thai–Myanmar border, and it was famous for excellent rice that won national awards for many years. The contamination of Cd in rice is associated with suspended sediment transported to fields via the irrigation supply. Over 90% of the rice grain samples collected contained Cd at concentrations exceeding the Codex Committee on Food Additives and Contaminants (CCFAC) draft Maximum Permissible Level for rice grain of 0.2 mg/kg. This poses a significant public health risk to local communities.

Prolonged consumption of the contaminated rice is believed to be a major source of Cd accumulation in the Mae Sot district residents. Our recent studies demonstrated that Mae Sot people have high risk of kidney damage with significantly high urinary Cd and high levels of renal dysfunction biomarkers such as α_1_- and β_2_-microglobulins (α_1_-MG, β_2_-MG) and N-acetyl-β-D-glucosaminidase (NAG) (Swaddiwudhipong et al. 2007; Teeyakasem et al. 2007; Honda et al. 2010). The residents also have high levels of Cd-associated chronic diseases such as hypertension, diabetes, osteoporosis and anemia (Swaddiwudhipong et al. 2010; Nambunmee et al. 2010,2011). Swaddiwudhipong et al. (2012) recently reported a five year study (2005–2010) of the residents with prolonged exposure to high levels of environmental Cd, showing toxic effects may persist long after Cd exposure is reduced.

Therefore, a sensitive, specific and economical method for early detection of renal dysfunction induced by Cd is needed for health monitoring of the population, in whom signs and symptoms of acute kidney injury may not yet be manifested. We report here a use of the KIM-1 as a sensitive biomarker for early detection of renal tubular dysfunction in Mae Sot residents with high Cd exposure and compared its prevalence with those of two conventional renal biomarkers, NAG and β_2_-MG concentrations to show usefulness of KIM-1 measurement among Cd exposed population at high level.

## Materials and methods

### Study population

Seven hundred residents permanently living in the Cd contaminated area in 12 villages in Mae Sot district, were recruited to this study. They were comprised of 260 men and 440 women aged between 21–89 years. They had been selected non-randomly from among those found in a 2004–2005 survey (Swaddiwudhipong et al. 2007) with high urinary Cd levels greater than 5 μg/g Cr. All subjects were advised of the aim and methods of the project and consented to provide morning urine sample, 5–10 ml of venipuncture blood, and demographic and health information. The study was approved by the Research Ethics Committee of the Faculty of Medicine, Chiang Mai University (Protocol approval No. 010/2009).

### Collection of urine and blood samples

The morning urine samples were collected in a polyethylene bottle after the subjects underwent physical examination and anthropometric measurements. Qualitative tests for pH, protein, glucose, occult blood, urobilinogen and ketone body were conducted on urine samples at the sampling site using paper strips (Ames test, Bayer, Germany). Each urine sample was divided into three aliquots of which any with pH < 5 one aliquot was adjusted to the pH 6–8 by 0.5N sodium hydroxide to prevent degradation of β2-MG in acidic urine. Venous blood was collected from each subject in an EDTA vacutainer. All samples were kept at −20°C before analysis.

### Determination of blood Cd and urinary Cd and creatinine

Blood and urinary Cd concentrations were quantified using a flameless atomic-absorption spectrometer (Shimadzu Model AAS-6300, Japan). The urine was diluted by 20 mg/l palladium chloride solution in 5%HNO_3_ as a matrix modifier at the ratio of 1:1. Proteins in blood were precipitated by 5%HNO_3_ at the ratio of 1:2 (Honda 2010). Method validation of the technique were performed and verified by certified standard reference materials [urine reference material Lot No. 2670 (National Bureau of Standards, Washington D.C.) and control blood Lot No. 620302 (Behring Institute, Dresden, Germany)], to ascertain the accuracy and precision of the method. Detection limits of urinary and blood Cd were 0.06 μg/g Cr and 0.2 μg/l, respectively. The urinary Cd concentrations of each subjects were adjusted by each urinary creatinine concentrations measured by the enzyme assay (Cica liquid–S, Kantokagaku Reagent Division, Ltd., Japan).

### Determination of urinary renal markers

The NAG level was quantified by a colorimetric assay using an NAG test kit (Shionogi Pharmaceuticals, Japan). The β2-MG level was quantified by an enzyme immunoassay (GLAZYME β2-microglobulin-EIA test kit, Sanyo Chemical Industries Ltd., Japan). Concentrations of both urinary NAG and β_2_-MG were also adjusted per g Cr.

The urinary KIM-1 was quantified by an in-house enzyme linked immunoassay (ELISA) using human KIM-1 standard, mouse anti-human KIM-1 antibody and horse radish peroxidase conjugated mouse anti-human KIM-1 antibody which were purchased from R&D system, Minneapolis, MN, USA. The technique of ELIZA development was validated according to a bioanalytical recommendation (FDA 2001; DeSilva et al. 2003). The detection limit of urinary KIM-1 was 32.2 pg/ml and the limit of quantification was 110.7 pg/ml. The detail process of preparation and validation of our KIM-1 assay was reported in somewhere (Panyamoon et al. 2009).

### Statistical analysis

Data analysis was performed using the SPSS statistical package (Version 11.5). Logarithmic transformation was applied to Cd concentrations in urine and blood and all renal markers to achieve a normal distribution before being subjected to data analysis. Correlations between Cd exposure markers and KIM-1 concentrations were assessed by Spearman’s rho analysis. Partial correlation analysis between Cd exposure and renal markers were performed after controlling for age and/or smoking habit at present. Comparisons of Cd exposure among groups at different smoking status and levels of renal markers among groups at different urinary Cd levels were analyzed by one-way ANOVA. Dose–response relationship between urinary Cd and KIM-1-uria was analyzed by probit regression model. At this time, KIM-1-uria was determined using cut-off value which was the 95 percentile values of urinary KIM-1 in the subjects with urinary Cd < 2 μg/g Cr (defined as non-exposure level of the urinary Cd by WHO). The P values of 0.05 or less were considered to identify statistical significance.

## Results

### Characteristics of the subjects and qualitative screening results

There were no different between genders of the average age, weight, height, body mass index and duration of residence in the area of the subjects in this study (Table 1). The mean age of the subjects was 54 years old and they all live permanently in the contaminated area for approximately 53 years (Table 1). Four hundred and twenty one subjects (61%) out of 700 (159 men and 262 women) were older than 50. Although all subjects had no severe clinical symptoms, the on-site screening examination with urinary strips showed 392 people had positive urinary protein, 326 subjects had positive blood, 2 subjects were found with positive urinary ketone and glucose and 105 subjects had urinary pH equal to 5 or lower. In addition, no participants had occupational exposure to Cd.

**Table 1:** **Characteristics of the subjects and concentrations of cadmium and renal markers**

Variables	Men	Women	Total
	(N = 260)	(N = 440)	(N = 700)
Age (years)	55.6 ± 14.0	53.1 ± 12.7	54.0 ± 13.2
Weight (kg)	54.7 ± 10.0	51.3 ± 10.6	52.6 ± 10.5
Height (cm)	160.7 ± 5.9	151.1 ± 5.1	154.6 ± 7.1
BMI (kg/m^2^)	21.1 ± 3.1	22.5 ± 4.2	22.0 ± 3.1
Duration of residence (years)	54.1 ± 15.0	52.5 ± 13.3	53.1 ± 14.0
Never smoked (N)	16 (6.2%)	216 (49.1%)	232 (33.1%)
Ex-smokers (N)	86 (33.1%)	118 (26.8%)	204 (29.1%)
Occasional smokers (N)	19 (7.3%)	20 (4.5%)	39 (5.6%)
Regular smokers (N)	139 (53.4%)	86 (19.6%)	225 (32.2%)
Blood cadmium (μg/L)	6.7 (1.0)	4.9 (2.0)	5.5 (2.0)
Urinary cadmium (μg/gCr)	6.3 (1.8)	7.0 (1.9)	6.7 (1.9)
KIM-1 (pg/gCr)	860 (2.3)	1050 (2.2)	974.8 (2.3)
NAG (U/gCr)	5.3 (1.9)	5.7 (1.9)	5.5 (1.9)
β2-MG (μg/gCr)	367 (9.5)	184 (6.1)	237.9 (7.5)

N: number of subjects; BMI: body mass index; μg/L: microgram per liter; μg/gCr: microgram per gram creatinine; pg/gCr: picogram per gram creatinine; U/gCr: unit per gram creatinine; KIM-1: Kidney injury molecule-1; NAG: N-acetyl-*β*-D-glucosaminidase; β2-MG: β_2−_microglobulin; values of cadmium and renal markers represent geometrical mean (geometrical standard deviation).

**Figure 1:**
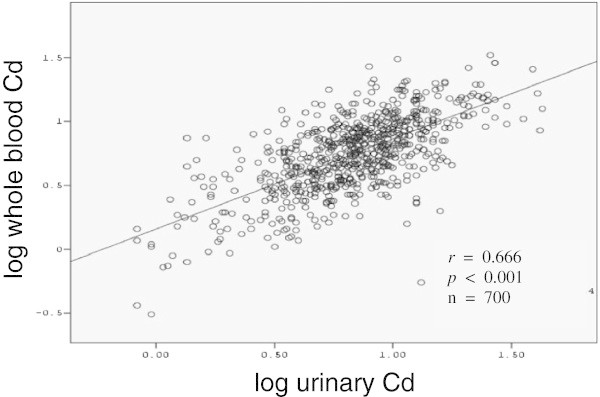
**Positive correlation of the cadmium concentrations in urine (μg Cd/g Cr) and whole blood (μg Cd/L) in the inhabitants (n=700) living in cadmium polluted area, Mae Sot district, Tak province (Spearman’s rho analysis).**

There were 232 subjects (33.1%) who had never smoked with lower rate found in men (6.2%) than in women (49.1%) and number of men with regular smoking (53.4%) were more than the number of regular smoking women (19.6%) (Table 1). The geometrical means of Cd in both urine and blood were also shown in Table 1 with no genders difference. Figure 1 showed scatter plot of blood Cd and urinary Cd with high correlation between them (r=0.666, P<0.001).

### Exposure markers, smoking status and renal dysfunction markers

Only blood Cd concentrations in smoking status were significantly different from those Cd levels in never smoked subjects in both men and women (Table 2). There was no age difference of urinary cadmium in both genders, however, blood Cd in aged group ≥ 50 years were significantly higher than those levels in the younger (P < 0.001, data not shown).

**Table 2:** **Cadmium exposure and smoking status of the subjects**

Smoking status	Blood Cd (μg/L)			Urinary Cd (μg/g Cr)		
	Men women total			Men women total		
Never smoked (n=232)	4.9 (2.0)	4.4 (2.1)	4.4 (2.1)	5.9 (2.3)	7.5 (3.0)	7.3 (3.0)
Ex-smoker (n=204)	6.3 (2.0)	4.8 (2.3)	5.4 (2.2)**	6.5 (2.0)	6.4 (2.0)	6.5 (2.0)
Occasional smoker (n=39)	7.1 (1.7)	6.8 (1.6)**	6.9 (1.6)***	7.8 (1.9)	7.1 (1.7)	7.5 (1.8)
Regular smoker (n=225)	7.3 (1.8)*	6.7 (1.6)***	7.0 (1.7)***	6.1 (1.8)	7.5 (1.6)	6.6 (1.7)

Cd: cadmium; μg/L: microgram per liter; μg/g Cr: microgram per gram creatinine.

*P<0.05; **P<0.01; ***p<0.001 compared to never smoked group.

Values of cadmium represent geometrical mean (geometrical standard).

**Figure 2:**
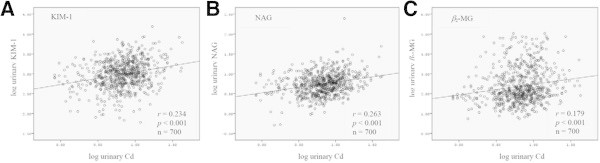
**Positive correlations of the urinary cadmium and renal dysfunction biomarkers; A) kidney injury molecule-1 (KIM-1), B) N-acetyl-β-D-glucosaminidase (NAG) and C) β2−microglobulin (β2-MG), using Spearman’s rho analysis.**

Scatter plots between log transformed urinary KIM-1, NAG and β2-MG and urinary Cd were shown in Figure 2. All of the renal markers were highly correlated to Cd exposure (P < 0.001). Then, unadjusted and adjusted correlations by age and smoking status between blood Cd and renal markers were performed because of significant relationship of blood Cd and smoking status, and their correlation coefficients were shown in Table 3. Urinary Cd was analyzed with simple and partial correlations after controlling age in comparison to renal markers (Table 3). Three renal biomarkers were significantly correlated with Cd exposure markers after adjusting age and/or smoking. Particularly, urinary KIM-1 was significantly increased (P < 0.001) with increase of blood Cd compared to the NAG and β2-MG in men after elimination of influence by age and smoking.

**Table 3:** **Relationships between cadmium exposure marker and renal dysfunction markers**

		KIM-1	NAG	β2-MG
Men	(N = 260)			
U-Cd	Corr.	0.295 ***	0.320 ***	0.182 **
	Partial corr.	0.289 ***	0.312 ***	0.156 *
B-Cd	Corr.	0.265 ***	0.200**	0.199 **
	Partial corr.	0.224 ***	0.150 *	0.132 *
Women	(N = 440)			
U-Cd	Corr.	0.215 ***	0.251 ***	0.190 ***
	Partial corr.	0.205 ***	0.242 ***	0.178 ***
B-Cd	Corr.	0.205 ***	0.165 ***	0.228 ***
	Partial corr.	0.161 **	0.103 *	0.174 ***

U-Cd: urinary cadmium; B-Cd: whole blood cadmium; N: number of subjects;

Corr.: correlation coefficient (Pearson); Partial corr.: partial correlation coefficient after controlling.

by age for U-Cd, by age and smoking habit (yes1, no0).

*p<0.05; **p<0.01; ***p<0.001.

### Dose effect and dose response relationships of renal dysfunction and urinary Cd

**Figure 3:**
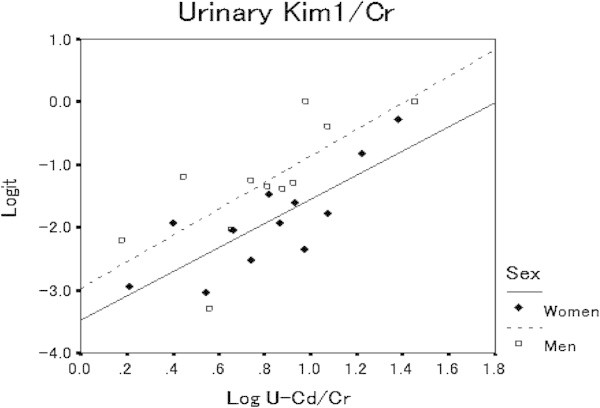
**Dose–response relationships between log10 transformed urinary cadmium concentrations (U-Cd) and logit-transformed prevalence rates of urinary KIM-1 (Logit) in men (open square) and women (black diamond).** Prevalence rates were determined using cutoff values for urinary KIM-1 at 1,577 and 2,413 μg/g Cr for men and women, respectively, which were 95 percentile values of urinary KIM-1 in men and women with urinary Cd < 2 μg/g Cr. Regression line: Y = 2.27 log X −3.12 for men (broken line), Y = 2.23 X −3.81 for women (solid line).

When 4 levels of urinary Cd concentrations were classified, the renal dysfunction biomarkers in this study show significant positive dose effect relationships with urinary Cd (Table 4). Moreover, to confirm the dose response relationships between urinary KIM-1 and urinary Cd, probit regression analysis was performed based on the prevalence rates of KIM-1-uria shown in Table 5. At this time, 95 percentile value of the quantified urinary KIM-1 in the subjects in both genders whose urinary Cd < 2 μg/g Cr was used as cutoff level to determine KIM-1-uria. After logit-converting prevalence of KIM-1uria, scatter plots between cadmium (dose) and prevalence rates of KIM-1-uria (response) were performed and shown in Figure 3, with probit regression lines of Y = 2.27X - 3.24 for men and Y = 2.23X - 3.81 for women. The regression lines demonstrates good fitness of Fit χ2 at 14.6 (P = 0.201) for men and 9.05 (P = 0.618) for women. When factor of age was added into the model, regression lines with good fitness were Y = 1.28X + 0.11Age – 8.56 (χ2 = 7.98, P = 0.631) for men and Y = 2.19X + 0.013Age – 4.48 (χ2 = 8.95, P = 0.537) for women. The estimated threshold value of the urinary Cd which caused KIM-1-uria and Cd concentrations corresponded to 10% prevalence of KIM-1-uria for the subjects aged 55.5 years old, was 1.3 μg/g Cr for men and 5.2 μg/g Cr for women.

**Table 4:** **Dose response relationship of the urinary cadmium (4 levels) and renal dysfunction biomarkers of the subjects**

Cadmium	N	KIM-1	NAG	β2-MG
(μg/g Cr)		(pg/g Cr)	(U/g Cr)	(μg/g Cr)
Men				
< 2	13	513.9 (2.2)	4.0 (2.2)	137.1 (5.0)
2–4.99	67	686.8 (2.2)	4.5 (1.8)	266.2 (9.4)
5–9.99	118	863.2 (2.2)*	5.0 (1.7)	345.0 (8.9)
≥ 10	62	1211.2 (2.1)**	7.5 (2.0)**	721.3 (10.5)*
Women				
< 2	23	711.9 (2.0)	3.8 (2.1)	61.4 (3.4)
2–4.99	95	922.4 (2.2)	5.1 (1.8)	175.1 (5.6)*
5–9.99	201	1040.9 (2.2)	5.5 (1.7)*	160.9 (5.9)*
≥ 10	121	1269.4 (2.4)**	7.1 (1.9)***	294.8 (6.7)***

N: number of subjects; KIM-1: kidney injury molecule-1; NAG: N-acetyl-*β*-D-glucosaminidase; β2-MG: β_2−_microglobulin.

*P<0.05; **P<0.01; ***P<0.001: significant different from the subjects group with cadmium concentrations below 2 μg/gCr (Dunnet T test for post hock).

Values of renal markers represent geometrical mean (geometrical standard deviation).

**Table 5:** **Prevalence rates of urinary KIM-1 among urinary Cd categories**

	U-Cd (μg/g Cr)	N	Mean of U-Cd	Mean of age (y)	KIM-1 prevalence (%)	
Men	0–0.99	3	1.0	45.0	0	(0.0)
	1–1.99	10	1.5	57.0	1	(10.0)
	2–2.99	13	2.8	51.0	3	(23.1)
	3–3.99	28	3.6	49.0	1	(3.6)
	4–4.99	26	4.5	51.5	3	(11.5)
	5–5.99	36	5.5	56.0	8	(22.2)
	6–6.99	34	6.5	53.0	7	(20.6)
	7–7.99	20	7.5	49.0	4	(20.0)
	8–8.99	14	8.4	58.0	3	(21.4)
	9–9.99	14	9.5	60.0	7	(50.0)
	10–14.99	47	11.8	61.0	19	(40.4)
	15–19.99	7	15.6	47.0	0	(0.0)
	20 –	8	28.4	56.0	4	(50.0)
Women	0–0.99	3	0.8	43.0	0	(0.0)
	1–1.99	20	1.6	52.0	1	(5.0)
	2–2.99	16	2.5	58.5	2	(12.5)
	3–3.99	44	3.5	50.5	2	(4.5)
	4–4.99	35	4.6	53.0	4	(11.4)
	5–5.99	41	5.5	51.0	3	(7.3)
	6–6.99	43	6.6	50.0	8	(18.6)
	7–7.99	40	7.3	53.0	5	(12.5)
	8–8.99	42	8.5	56.0	7	(16.7)
	9–9.99	35	9.4	54.0	3	(8.6)
	10–14.99	77	11.7	54.0	11	(14.3)
	15–19.99	23	16.5	52.0	7	(30.4)
	20 –	21	23.9	55.0	9	(42.9)

N: number of subjects, y: years, U-Cd: urinary Cd.

Cutoff values for U-KIM-1 = 1,577 μg/g Cr for men, 2,413 μg/g Cr for women, which were 95%tile values of the subjects with U-Cd < 2 μg/g Cr.

## Discussion

Mae Sot district was the first reported case of environmental Cd in Thailand from zinc mining activities. The Cd contaminated area was estimated to be about 13,200 rais (× 1,600 m^2^) of paddy field affecting 12 villages with a total population of 12,075 in 2004 (Data from Tak Provincial Office). Since the residents consumed local rice grown daily, they were at high risk of chronic Cd toxicity. Health risk assessment and biomarkers monitoring among residents is important to help protect systemic toxicity of Cd before reaching severity. Among our selected subjects, we found 36 people have urinary Cd lower than 2 μg/g Cr and 183 subjects (62 men and 121 women) with very high urinary Cd over 10 μg/g Cr, indicating that the exposure is still going on in some subjects and some but less has reduced. One of the subjects had urinary KIM-1 at the concentration of 15330 pg/g Cr, suggesting the existence of severe renal tubular injury in Mae Sot residents. This person was later referred for medical checkup and proper healthcare at Mae Sot General Hospital.

Blood and urinary Cd are useful indicators of Cd body burden, whereas blood Cd also reflects Cd exposure during recent several years (WHO 1992). Men were found to have statistically significant higher blood Cd concentrations than women (Table 2). The results were different from a previous report of Rey et al. (1997) in which there was no difference between gender and blood Cd concentration was 0.94 μg/L in men and 1.12 μg/l in women. Willer (1992) also reported the concentration of whole blood Cd at 0.19 μg/L. The difference in these studies might have resulted from either high dietary Cd exposure or smoking consumption. Since smoking is one of cadmium exposure source, Cd concentrations in blood and urine with never smoked people showed high Cd levels over 2 μg/g Cr and blood Cd was increased accordingly to the smoking status (Table 2), however, urinary Cd concentrations were not different between non-smokers and smokers. Therefore, correlations of blood Cd with renal markers were analyzed after adjusting not only age but also smoking status, but correlations of urinary Cd was computed after adjusting only age.

The critical concentration of Cd that induced nephropathy has been studied. Because nephropathy was not a clear-cut clinical entity, many biomarkers for early detection of Cd induced renal dysfunction such as NAG, albumin, β_2_-MG, α_1_-MG etc. had been used in several studies (Jung et al. 1993; Jin et al. 2002; Moriguchi et al. 2003; Teeyakasem et al. 2007). In Thai subjects, Teeyakasem et al. (2007) used urinary Cd as an index of Cd exposure and used β_2_-MG, NAG, total protein, albumin, aminonitrogen, lysozyme and glucose as indicators of Cd nephrotoxicity. However, another low molecular weight protein, i.e. kidney injury molecule-1 (KIM-1) had not yet been studied in the urine of high Cd exposure such as that in the Mae Sot inhabitants. The developed in house-ELISA for quantitating urinary KIM-1 validated in our previous study (Panyamoon et al. 2009) was highly sensitive, with LOD of 33.2 pg/mL and LOQ of 110.7 pg/mL. There was no urinary sample with KIM-1 concentration lower than the LOQ value. The assay precision in all %CV was less than 20% and was accepted as very high precision (FDA 2001). The assay accuracy was also high with approximately 90% recovery. This result indicated that our developed ELISA technique gave a very high sensitivity and specificity for KIM-1 measurement in urine samples. In addition, KIM-1 in urine was stable even after the urine was frozen and thawed for 4 cycles. The finding was similar to what Han and Bonventre (2004) reported. KIM-1 was also stable for short-term storage at 4°C for 5 days. Nowadays, there is limited data of KIM-1. Urinary KIM-1 had been mostly used as a biomarker of nephrotoxic injury in animal models (Ichimura et al. 1998; Vaidya et al. 2006; Prozialeck et al. 2009) and only in human patients with renal diseases (Han et al. 2002). This is the first report of KIM-1 as a potential marker on indication of renal dysfunction induced by environmental exposure to cadmium.

Urinary excretion of NAG had been recommended as a sensitive biomarker of Cd exposure and is more sensitive than β_2_-MG (WHO 1992). However, Jung et al. (1993) recommended a combination of NAG and α_1_-MG for early detection of Cd induced renal injury. Fifty eight men and 123 women in this study had urinary NAG excretion over 8 U/g Cr suggested proximal tubular damage had occurred. Although the urinary NAG was shown to be the best biomarker that correlated well with urinary Cd, we found that the prevalence rate of NAG-uria was lower than hyper KIM-1-uria. In addition, an increase of NAG level had been reported in variable of conditions such as chronic glomerular disease (Bazzi et al. 2002) and diabetic nephropathy (Ikenaga et al. 1993). The urinary NAG is quantitated by colorimetric assay technique and its activity might be inhibited by endogenous urea (Bondiou et al. 1985), while urinary KIM-1 was measured via antigen-antibody reaction; therefore, KIM-1 might be more sensitive than urinary NAG.

In this study, urinary β_2_-MG had wide ranges between 5–107,883 μg/g Cr. Two women had β_2_-MG concentrations over 100,000 μg/g Cr, suggesting severe renal tubular dysfunction. However, an increase of urinary β_2_-MG excretion had been also found in other diseases, cardiac surgery (Dehne et al. 1995) and renal transplantation (Schaub et al. 2005). In addition, urinary β_2_-MG likes to be degraded rapidly at room temperature and in the urine with pH less than 6.0. Thus, it is reasonable to conclude that urinary KIM-1 is a better biomarker for indicating renal tubular dysfunction than β_2_-MG.

In the present study, clear dose–response relationship between urinary KIM-1 and urinary Cd was shown, and threshold values of KIM-1-uria in both genders were lower than those of urinary NAG and β_2_-MG. These results also confirm urinary KIM-1 is useful marker to detect urinary tubular dysfunction at early stage. However, in this analysis, 95 percentile values of KIM-1 of men and women with urinary Cd < 2 μg/g Cr were used for cut-off values to calculate of prevalence of KIM-1-uria. This is a big limitation of this analysis, because we have no controls and residents with lower exposure needed to use as controls. In future, we select suitable controls for Mae Sot residents and measure KIM-1 to detect reference value for Thai population. Then, we are going to use this marker to detect early sign of renal effect in Cd exposed subjects, particularly young people with low Cd body burden.
